# Emerging interactions between matrix components during biofilm development

**DOI:** 10.1007/s00294-015-0527-5

**Published:** 2015-10-29

**Authors:** David E. Payne, Blaise R. Boles

**Affiliations:** Department of Microbiology, Roy J. and Lucille A. Carver College of Medicine, University of Iowa, Iowa City, IA USA

**Keywords:** *Staphylococcus aureus*, Functional amyloids, Biofilm, eDNA, Polysaccharide

## Abstract

Bacterial cells are most often found in the form of multicellular aggregates commonly referred to as biofilms. Biofilms offer their member cells several benefits, such as resistance to killing by antimicrobials and predation. During biofilm formation there is a production of extracellular substances that, upon assembly, constitute an extracellular matrix. The ability to generate a matrix encasing the microbial cells is a common feature of biofilms, but there is diversity in matrix composition and in interaction between matrix components. The different components of bacterial biofilm extracellular matrixes, known as matrix interactions, and resulting implications are discussed in this review.

## Biofilms and their matrix

A common strategy employed by bacteria to survive in varied environmental conditions is to develop into an encased community of cells called biofilm. The fact that these multicellular communities can grow on surfaces with diverse chemistries, persist in hostile environments, and resist clearance by strategies that typically eradicate planktonic bacteria is a topic of much interest to researchers from diverse disciplines. It is clearly evident that many pathogenic bacteria use the biofilm growth mode to persist in the host and avoid clearance by the immune system and antimicrobial chemotherapies leading to the development of persistent infections (Costerton et al. 1999; Mathe and Van Dijck 2013; Parsek and Singh 2003). The increasing use of implant materials in healthcare settings further exacerbates this problem as foreign bodies are known to promote the initiation of biofilms (Thurlow et al. 2011; Zimmerli et al. 2004).

A major area of interest in biofilm research is the composition of the substances holding these communities of cells together. This polymeric material is usually referred to as the “biofilm matrix.” The exact composition, potential interactions, and the role of the different matrix components are not fully understood. There is considerable interest in gaining an improved understanding of the biofilm matrix because bacteria with matrix defects are often unable to form a biofilm and treatments that breakdown the matrix transition cells to an antimicrobial susceptible state (Boles and Horswill 2008, 2011; Lauderdale et al. 2010; Payne et al. 2013). The exact composition of the biofilm matrix can vary greatly between different bacterial species, strains, and growth conditions, therefore, it should be noted that not all biofilms are equivalent and variation has routinely been observed between strains of the same species.

**Fig. 1:**
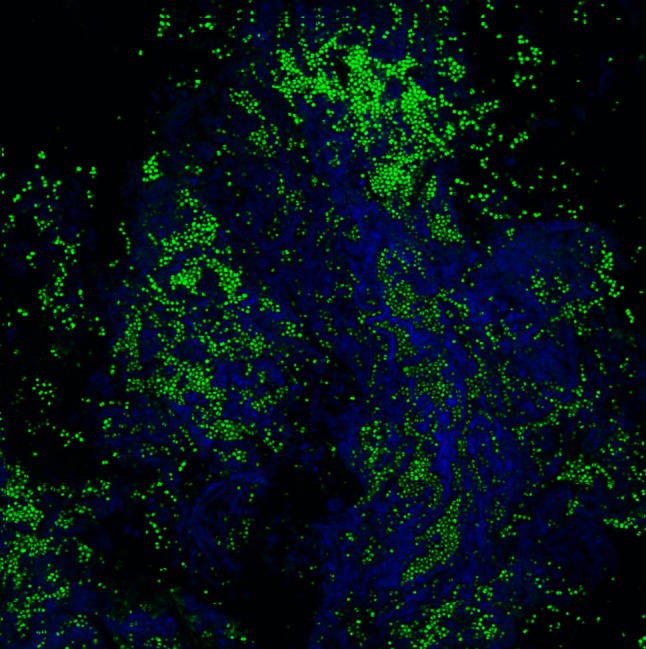
Confocal micrograph of a *Staphylococcus aureus* biofilm growing on a catheter. The biofilm was stained with two DNA strains, syto-9 (*green*) and draq-5 (*blue*). Green cocci are *S. aureus* cells encased in a blue matrix that consists part of eDNA

In the case of *Staphylococcus* species, including *S. aureus*, the primary matrix components consist of polysaccharides (Cramton et al. 1999), proteins (Beenken et al. 2010; Cramton et al. 1999; Lauderdale et al. 2010; Marti et al. 2010; O’Neill et al. 2008; Tsang et al. 2008), and extracellular DNA (eDNA) (Izano et al. 2008; Kaplan et al. 2012) (Fig. 1). Though not commonly appreciated as matrix components, extracellular teichoic acids have been purified from the matrix material of Staphylococcal biofilms (Chaignon et al. 2007) and peptidoglycan has been proposed to play an unidentified role in the matrix based on observations that exposure to lysostaphin, an enzyme capable of degrading the pentaglycine bridge in the staphylococcal cell wall, can successfully detach biofilms (Kokai-Kun et al. 2009). In addition, host factors are likely to be incorporated into the biofilm matrix but this is largely unstudied and likely varies depending upon infection site. Below, we attempt to summarize much of what is known about these matrix components and their potential interactions.

## Polysaccharide

Many Staphylococcal species produce a poly-*N*-acetyl glucosamine (PNAG) polysaccharide, which is also referred to as polysaccharide intercellular adhesin (PIA). PNAG is synthesized by enzymes encoded by the *ica* operon and deposited on the cell wall surface (Cramton et al. 1999). Many environmental growth conditions likely contribute to the role of PNAG in the *Staphylococcus* biofilm matrix. These include anaerobic growth, high temperatures, osmolarity, and other environmental stresses including the presence of sub-inhibitory concentrations of antibiotics (O’Gara 2007). In some animal models of Staphylococcal biofilm infections, PNAG has been shown to play a crucial role in establishing a biofilm and maintaining a persistent infection (Rupp et al. 1999a, b, 2001). Because of this role in pathogenesis, efforts have been made to utilize PNAG as a vaccine candidate and have been met with mixed results. More recent evidence suggests that while some Staphylococcal strains rely on polysaccharides for robust biofilm formation, others form polysaccharide-independent biofilms (Boles et al. 2010; Izano et al. 2008; O’Gara 2007; Rohde et al. 2007). In cases of polysaccharide-independent biofilm formation, proteins and eDNA most likely substitute for PNAG as a structural matrix component.

## Proteins

Proteins are another major biofilm matrix component, as evidenced by the susceptibility of staphylococcal biofilms to proteases (Beenken et al. 2010; Boles and Horswill 2008; Marti et al. 2010). Many cell–cell and cell-host tissue contacts within a biofilm are mediated by surface proteins. Some surface proteins, such as the fibronectin binding proteins (O’Neill et al. 2008), protein A (Merino et al. 2009), SasG (Conrady et al. 2008, Corrigan et al. 2007), and biofilm associated protein (BAP) (Trotonda et al. 2005), have been defined as being important in cell–cell and cell–surface interactions occurring during biofilm development. It has also been suggested that cytoplasmic proteins play a moonlighting role in the matrix where they associate with cells upon a drop in pH (Ebner et al. 2015; Foulston et al. 2014). In addition, amyloids have recently emerged as an important proteinaceous component of many microbial biofilms, including *S. aureus*. First identified in human neurodegenerative diseases, amyloids are insoluble fibrous aggregates of proteins that contain parallel beta sheets. The amyloid fibers produced by *S. aureus* are composed of small peptides called phenol-soluble modulins (PSMs) (Schwartz et al. 2010, 2012, 2014). Amyloids are notorious for being relatively resistant to protease digestion and insoluble in detergents. Therefore, in the biofilm environment, these amyloids offer resistance to proteases and surfactants capable of degrading biofilms.

## eDNA

Extracellular genomic DNA (eDNA) is thought to be an important structural component in many bacterial biofilms including those formed by *S. aureus* (Okshevsky and Meyer 2014, 2015; Vorkapic et al. 2015). The addition of DNase to growing or mature biofilms of various bacterial species results in inhibition of biofilm formation or disruption of the established biofilms. However, DNase-mediated disruption of established biofilms is dependent upon biofilm age with “young” biofilms being more sensitive than “old” biofilms. In *S. aureus*, autolytic activity from a subpopulation of cells results in the release of genomic DNA that contributes to cell adhesion during biofilm maturation (Qin et al. 2007). The chemical nature of the long charged DNA molecule is thought to modulate the cell surface properties and to promote cell-to-cell and cell-to-surface adhesion and to serve a structural role in the *S. aureus* biofilm matrix (Mann et al. 2009; Rice et al. 2007).

## Matrix interactions

Several recent studies have emerged, suggesting that matrix components are capable of interacting and influencing biofilm development. Structural analysis revealed that the secreted *S. aureus* protein beta toxin has a three-dimensional structure that resembles nuclease. Although it lacks nuclease activity, it is capable of binding to eDNA, and oligomerizing to form higher ordered states. The beta toxin–eDNA interconnection acts as a skeletal framework of the biofilm that is critical for the biofilm matrix during infection (Huseby et al. 2010). The fact that many clinical isolates possess a phage interrupting the beta toxin gene has lead to the thought that this protein is not relevant to infection in those strains and there may be other eDNA-binding proteins that await identification. However, this notion has recently been challenged by work showing phage excision can occur during infection resulting in active beta toxin production (Salgado-Pabon et al. 2014).

Cytoplasmic proteins presumably released via autolysis have also recently been shown to interact with eDNA in a pH-dependent manner and this inaction offers protection from matrix degrading enzymes (Dengler et al. 2015). This work suggests that rather than dedicated proteins being made to contribute to the biofilm matrix, cytoplasmic proteins may “moonlight” in the matrix environment offering interactions between eDNA and cell wall material. Indeed, in gram-negative pathogens such as *Haemophilus influenza* and *Burkholderia cenocepacia,* proteins such as the integration host factor (IHF) protein have been shown to interact with eDNA and stabilize biofilms. Attempts to remove these proteins from biofilms using antisera that block protein–DNA interactions have shown promise at reducing biofilm biomass in vitro and treating chronic infections in animal models (Goodman et al. 2011; Jurcisek and Bakaletz 2007; Novotny et al. 2013). Considering that IHF is a member of the DNABII protein family that includes nucleoid-associated proteins like HU, which are present in both gram-negative and gram-positive pathogens, this strategy may hold promise at reducing biofilms against a variety of bacterial species. Recent work in *S. aureus* has also suggested that proteins typically thought of as being cytoplasmic could have a role in the biofilm matrix (Dengler et al. 2015; Foulston et al. 2014). The authors found the association of eDNA with the biofilm matrix was dependent on matrix proteins, some of which seem to have a moonlighting role in the matrix, as they are cytoplasmic proteins only released from the cell upon autolysis (Dengler et al. 2015). The addition of eDNA to DNase-treated cells could rescue biofilm formation/clumping suggesting a role for eDNA in facilitating cell-to-cell interactions.

Amyloids have also been shown to interact with other matrix components in the biofilm matrixes of *S. aureus* and *E. coli* (DePas and Chapman 2012). In the case of *S. aureus*, it was found that the presence of eDNA promotes the polymerization of amyloidogenic peptides (phenol -soluble modulins (PSM)) at concentrations that PSMs alone do not readily polymerize (Schwartz et al. 2015). It is suggested that this is a result of DNA attracting the positively charged PSMs and raising the local peptide concentration, therefore resulting in polymerization. In *E. coli* the functional amyloid component, CsgA has been shown to bind to DNA, promoting curli amyloid assembly (Fernandez-Tresguerres et al. 2010) and the resulting DNA/amyloid complex acts to stimulate autoimmunity (Gallo et al. 2015).

Interactions between eDNA and polysaccharides have also been observed in biofilms. In *P. aeruginosa*, two main biofilm matrix components (eDNA and the polysaccharide Psl) cooperate by physically interacting in a biofilm to form the web of Psl–eDNA fibers, which functions as a skeleton to allow bacteria to adhere and grow (Wang et al. 2015). Psl can interact not only with DNA of *P. aeruginosa*, but also the genomic DNA from human neutrophils and *S. aureus*, implying that *P. aeruginosa* has the ability to use DNA of other organisms to form its own communities.

## Outlook

Despite the importance of microbial biofilms to human health and industrial processes interactions between biofilm matrix components, remain poorly defined. Emerging work discussed above is demonstrating several matrix interactions (eDNA–protein, eDNA–amyloid and eDNA–polysaccharide) and there is little doubt that more interactions between matrix components and the mechanism underlying these interactions await to be elucidated. Interactions between matrix components within the biofilm are likely responsible for creating an adaptable structure during adherence, maturation, and dispersal. These findings underscore the notion that the formation of biofilm matrix is a complex, dynamic process with contribution of multiple factors, including bacterial cell death, the release of eDNA, the secretion of protein and the interaction between the matrix components.

In future work, it will be of interest to understand how host factors may be incorporated into bacterial biofilm matrixes and how these potential interactions may influence biofilm development. Considering that the negatively charged polymer DNA is central to most known matrix interactions, it is tempting to speculate that negatively charged host polymers such as hyaluronic acid and heparin could play a similar role. Biofilm matrix interactions may also provide novel targets for disrupting biofilm formation and eradicating established biofilms. Considering the dire health consequences many biofilm infections impose, this should be an area of emerging interest.
